# Effect analysis of iliac bone autografting for Hepple V osteochondral lesions of the talus

**DOI:** 10.1186/s13018-022-02924-w

**Published:** 2022-01-15

**Authors:** Xin Wang, Dong Zhang, Fengqi Zhang, Lin Jin, Donglin Shi, Zhiyong Hou

**Affiliations:** 1 Department of Orthopaedic, The Third Hospital of Hebei Medical University, No. 139 Ziqiang Road, Shijiazhuang, 050051 Hebei People’s Republic of China; 2 Division of Sports Science and Physical Education, Tsinghua University, Beijing, People’s Republic of China; 3 Hebei Sport University, Shijiazhuang, Hebei 050041 People’s Republic of China

**Keywords:** Iliac bone, Hepple V, Osteochondral lesions, Talus

## Abstract

**Background:**

Talar cartilage injury is a kind of disease that causes long-term and chronic pain of ankle joint. Autologous osteochondral transplantation has been viewed as an alternative choice for treating these lesions, but donor-site morbidity has limited its application. This study aimed to analyze the efficacy of iliac bone autografting for Hepple V osteochondral lesions of the talus.

**Methods:**

This retrospective study included 32 patients surgically treated for Hepple V osteochondral lesions of the talus from January 2015 to January 2020. All patients underwent open surgery. Ipsilateral iliac bone grafts were taken and filled with talar cartilage injury area. The improvement of postoperative ankle pain was evaluated by Visual Analogue Scale (VAS), and the improvement of ankle function was evaluated by the American Orthopaedic Foot & Ankle Society (AOFAS). During the postoperative follow-up, X-ray examination of the front and side of the ankle joint and CT of the ankle joint were performed to evaluate the bone cartilage healing in the graft area.

**Results:**

Thirty-two patients (32 ankles) (100%) returned for clinical and radiologic follow-up at an average of 28 (range 24–36) months postoperatively. At 3 months postoperatively and at the last follow-up, the AOFAS scores were (80.4 ± 3.6) and (89.2 ± 6.4), respectively, which were significantly improved compared with the preoperative score (49.7 ± 8.1), and the difference was statistically significant (*P* < 0.05). The VAS scores were (2.1 ± 0.9) and (1.5 ± 0.8), respectively, which were significantly better than the preoperative score (6.2 ± 1.7), and the difference was statistically significant (*P* < 0.05). Re-examination of the front and side of the ankle joint X-rays and CT showed that the bone healing at the osteotomy of medial malleolus and osteochondral transplantation area. All patients had no pain at the donor site. No complications occurred in 32 patients at the last follow-up.

**Conclusions:**

With iliac bone autografting for Hepple V osteochondral lesions of the talus can effectively relieve ankle joint pain and significantly improved ankle function.

**Level of evidence:**

Level III, Retrospective series.

## Introduction

Osteochondral lesion of the talus (OLT) is an important cause of ankle pain and functional limitation [1]. From the anatomical view, ankle site had approximately 1/2–3/5 the maximum cartilage thickness at weight-bearing areas as that of hip and knee (2.7 mm vs. 3–6 mm) [2]. Thus, any abnormal alignment of the ankle would lead to local stress concentration and accelerate the degeneration of ankle cartilage [2, 3]. Many studies have shown that patients with repeated sprains or fractures of the ankle have a high probability of talar cartilage injury [4, 5]. In the early stages of talar cartilage injury, there are no obvious signs and the pain is diffuse and poorly localized; in the late stages, the main clinical manifestations are ankle swelling and pain (aggravated during weight-bearing walking) and joint strangulation. The two common types of OLT are anterolateral and posteromedial. Anterolateral OLT occurs in about 42% of cases and has an acute onset, mostly due to inversion sprain of the ankle during dorsiflexion, resulting in an oval shallow lesion; posteromedial OLT (about 58%) is caused by repetitive microtrauma or overload, mostly due to inversion sprain during plantarflexion, resulting in a cup-shaped deep lesion [6–10].

Currently, there is well-established consensus that conservative treatment is effective for early talar cartilage damage (Stage I and II defined by Hepple classification), which typically imaging presents with injury of the articular cartilage alone [1]. The conservative treatment is mainly by reducing physical activity, avoiding vigorous exercise, and taking appropriate rest; intraarticular injection of platelet-rich plasma (PRP) [11], extracorporeal shock wave therapy, and oral medications may also be administered [7]. However, there is controversy regarding the treatment of talus cartilage injuries of stage III–V, especially Hepple V; for doctors and patients, there are great challenges in the treatment process. At present, the main surgical approaches include autologous or allogeneic osteochondral transplantation, and autologous chondrocyte implantation or periosteal bone grafting [12], and arthroscopic microfracture or drilling or arthroscopic autologous matrix-induced chondrogenesis for the treatment of articular cartilage defects of the talus [13, 14]. Although arthroscopic drilling has the advantages of minimal trauma and rapid recovery, it is only effective for lesions < 15 mm [15]; long-term follow-up studies show that bone cysts deteriorate after minimally invasive surgery, leading to surgical failure and poor results, making it unsuitable for larger cartilage injuries [16, 17]. Osteochondral grafting using autologous, allogeneic, or engineered bone grafts is becoming widely used for osteochondral injuries, especially for Hepple V OLT. Allogeneic osteochondral grafts avoid donor complications, but carry the risks of graft rejection and disease transmission. Autologous osteochondral grafts are usually taken from the osteochondral column of the ipsilateral knee, producing hyaline cartilage with excellent biomechanical properties, but potentially leading to irreversible knee damage. However, iliac bone grafts have fewer complications in the treatment of talar cartilage injuries, and the iliac periosteum has multifunctional stem cells with the ability to differentiate into chondrocytes and fibrocartilage [8, 9, 18].

Considering the increasing importance of evidence-based data on decision-making for the surgical option for this increasingly growing arthropathy in the patients, it is necessary to clarify the advantages and disadvantages of surgical methods to facilitate the effective management. Given that, we designed this study, with the aim of analyzing the efficacy of iliac bone autografting for osteochondral lesions of the talus with subchondral cysts in patients with Hepple V, in terms of pain relief, functional recovery, and postoperative complications.

## Materials and methods

This was a retrospective study. The study protocol was approved by the institutional review board of the Third Hospital of Hebei Medical University, and all patients provided the written informed consent.

The inclusion criteria were age > 18 years; chronic pain and swelling of the medial malleolus for > 3 months and failure of conservative treatment; Hepple V medial talar cartilage lesion on MRI; no prior talar surgery; good physical condition and ability to tolerate surgery; and provision of written consent for postoperative follow-up.

The exclusion criteria were the cartilage injury in other locations, such as the lateral or central part of the talus; severe ankle arthritis; active infection of the ankle; history of talar fracture; other serious deformities or diseases of the foot and ankle, such as clubfoot or diabetic foot; inability to tolerate surgery due to serious disease; and unwillingness to cooperate with postoperative treatment or follow-up.

The above criteria identified 32 consecutive patients who underwent iliac bone grafting for treatment of medial OLT with subchondral cysts after failure of conservative treatment from January 2015 to January 2020. The cohort comprised 19 men and 13 women (mean age 35.8 years; range 25–59 years), with 17 left-sided injuries and 15 right-sided injuries. All patients had posteromedial cartilage injury, including seven patients engaged in sports activities, five workers who were required to stand for prolonged periods of time, 11 manual workers, eight patients with a sedentary lifestyle that lacked physical activity, and two with other conditions. All patients underwent preoperative MRI and CT examination of the ankle, including measurements of the diameter and area of the cystic lesion. According to the American Orthopaedic Foot & Ankle Society (AOFAS) and the Visual Analogue Scale (VAS), the ankle joint function and the severity of ankle joint pain were evaluated and recorded in all patients.

### Surgical procedure

**Fig. 1:**
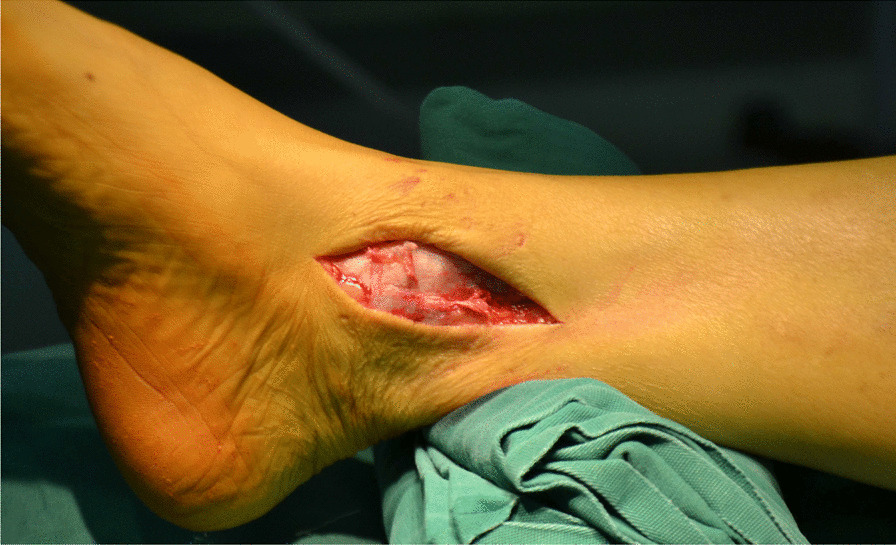
A 5-cm-long curved incision was made on the anteromedial side of the affected ankle

**Fig. 2:**
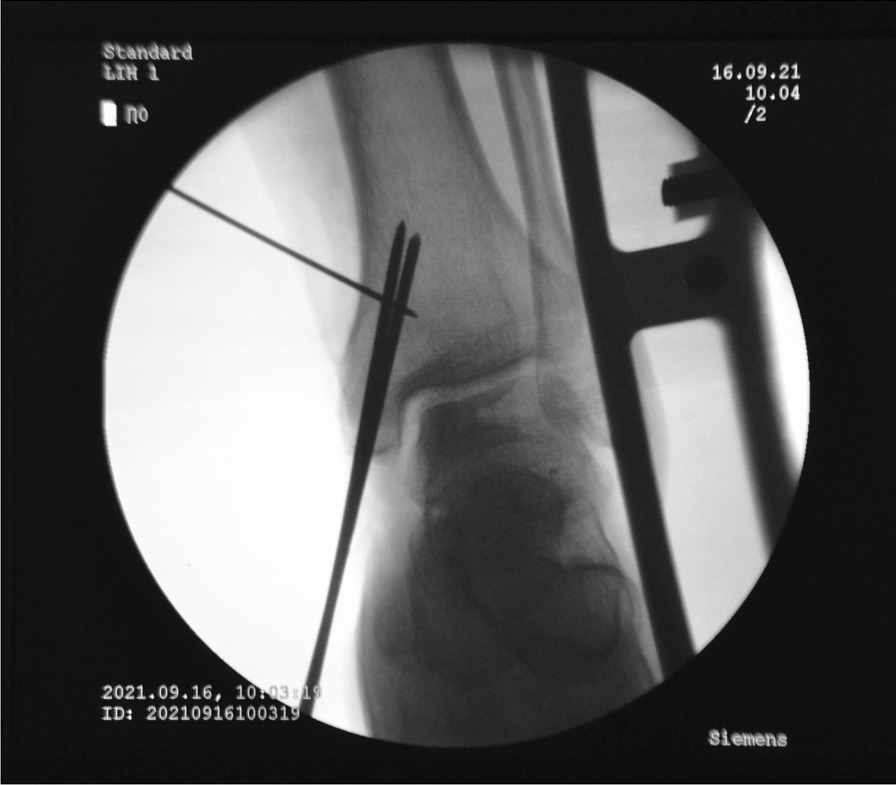
Two 3-mm-diameter Kirschner wires were used to enter the tibia at a 45° angle, and about 2 cm above the tip of the medial malleolusone, one 2.5 mm Kirschner wire was used to enter the tibia and remained vertical to the previous Kirschner wire. X-ray fluoroscopy showed that the position was satisfactory

**Fig. 3:**
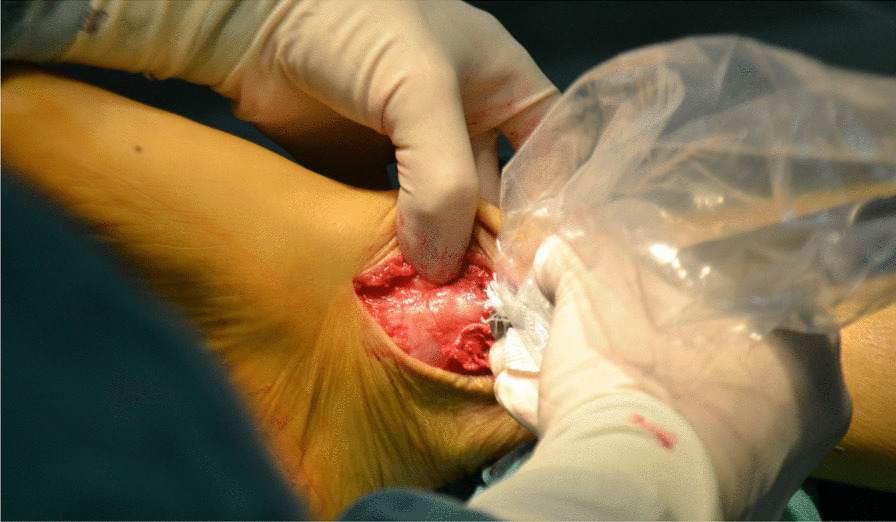
The swing saw was used to cut off the medial tibia along the direction of 2.5 mm Kirschner wire

**Fig. 4:**
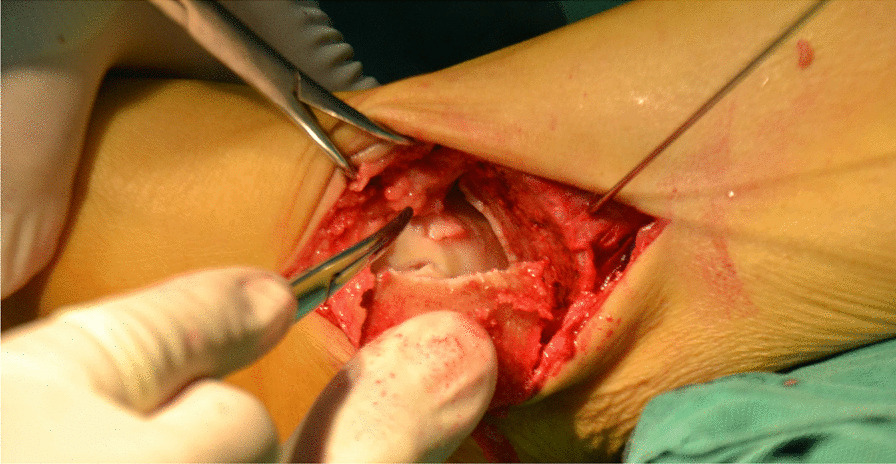
The valgus ankle joint fully exposed the cartilage damage area on the medial side of talus fornix

**Fig. 5:**
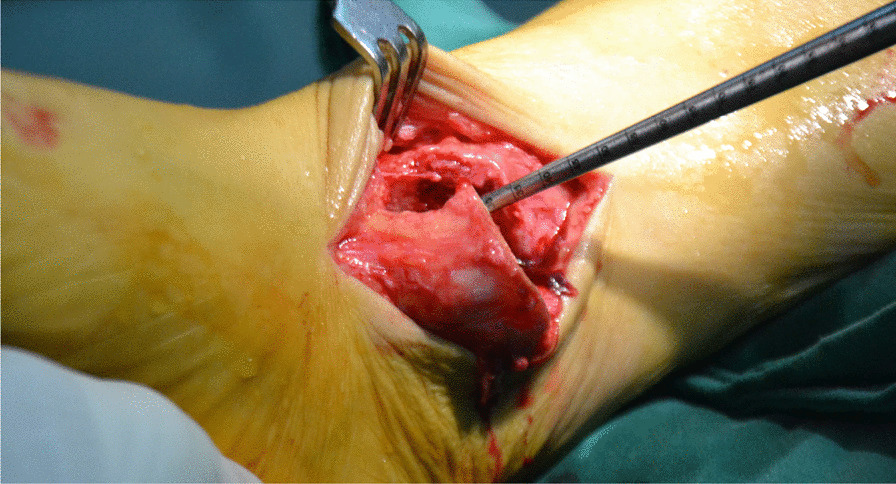
The cartilage scraper was used to remove the degraded cartilage

**Fig. 6:**
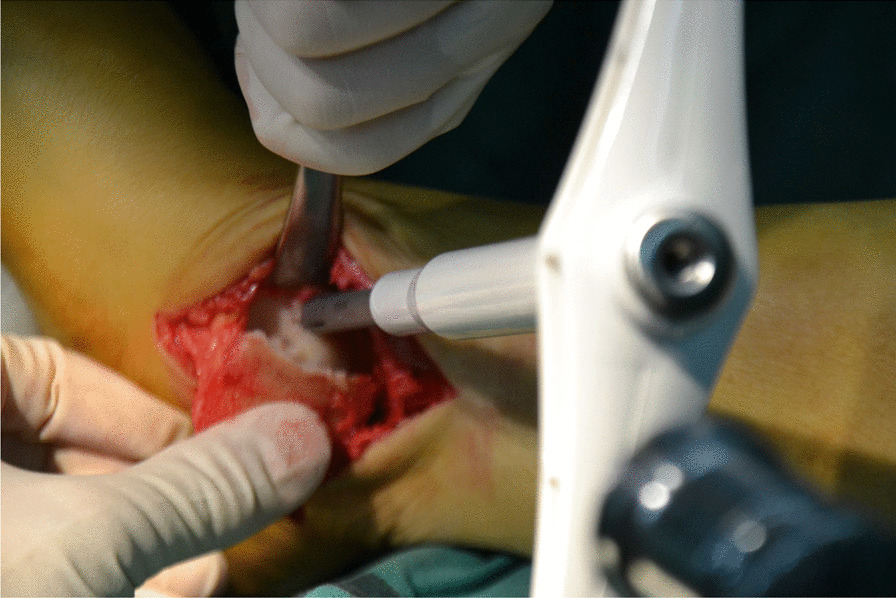
A bone extractor was used to punch a vertical hole in the joint surface and remove the lesion and cyst

**Fig. 7:**
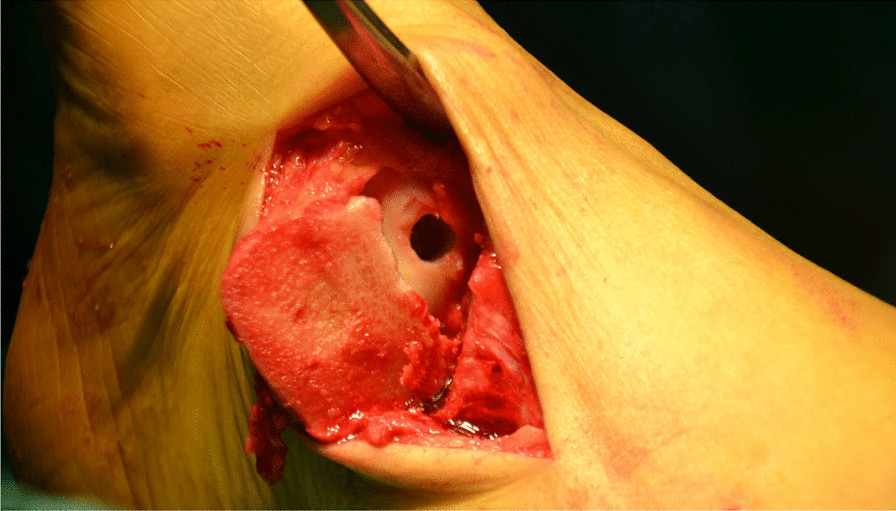
A cavity is visible after removing the talus necrotic tissue

**Fig. 8:**
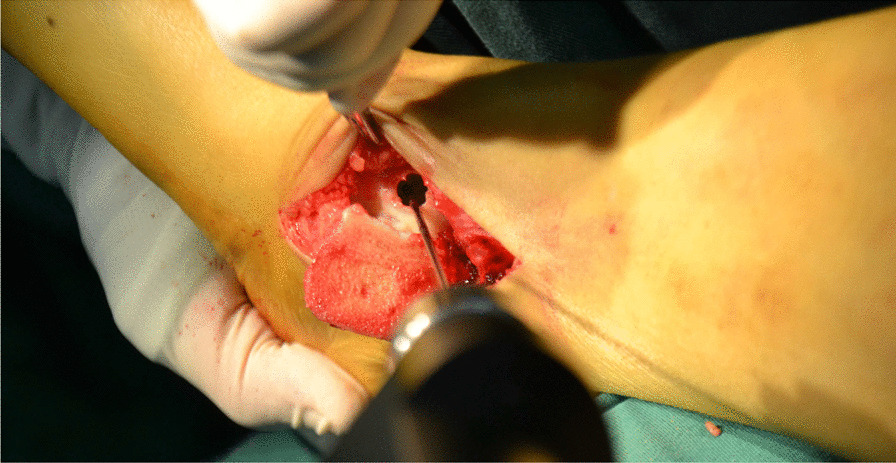
Kirschner wire was used to make microfractures in the sclerotic bone around the cystic cavity, and take uniform blood infiltration as the standard

**Fig. 9:**
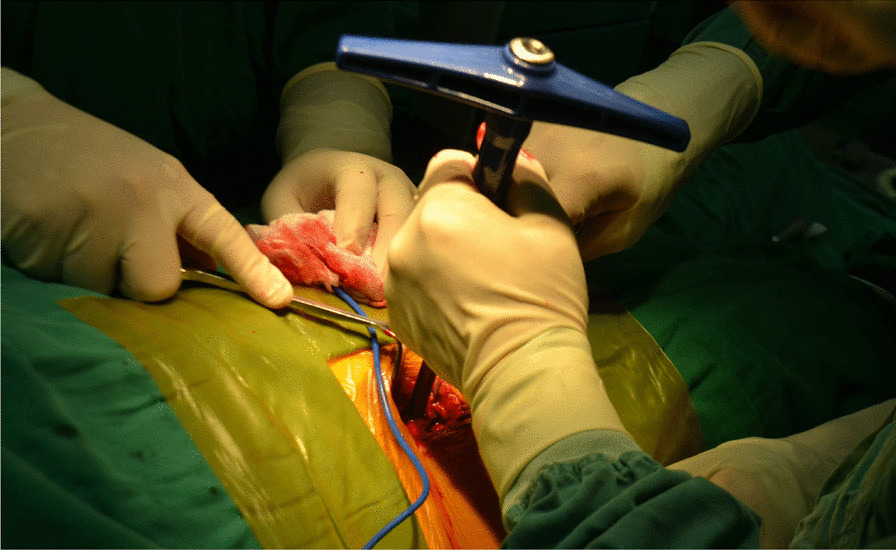
The bone extractor is used to remove the iliac composite bone column

**Fig. 10:**
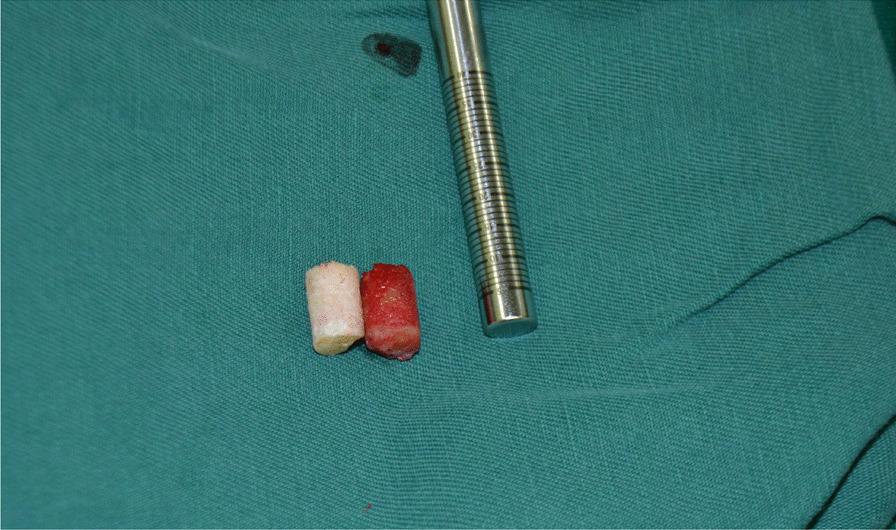
Comparison of iliac bone graft and talus necrosis bone, talus necrosis bone is white, lack of blood supply, ilium is bright red, and blood supply is abundant

**Fig. 11:**
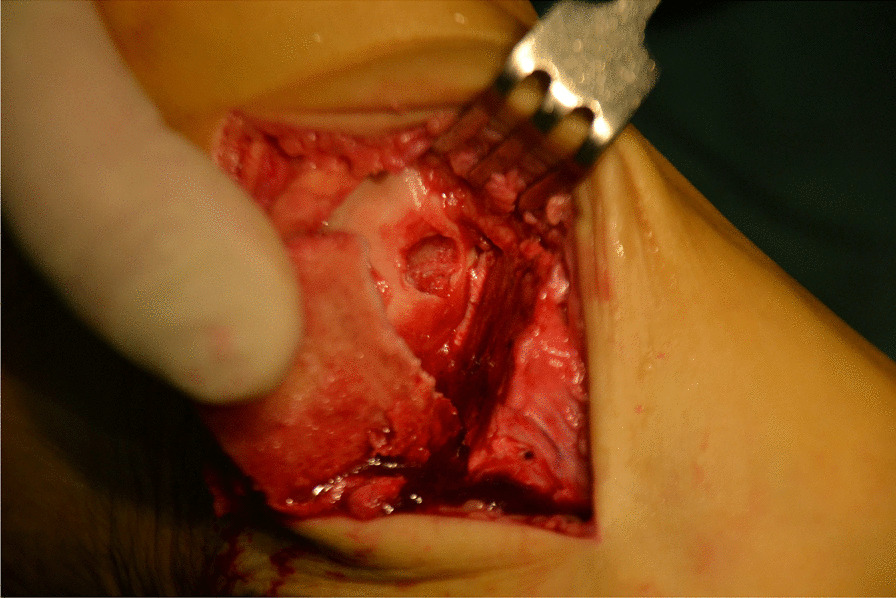
After bone grafting

**Fig. 12:**
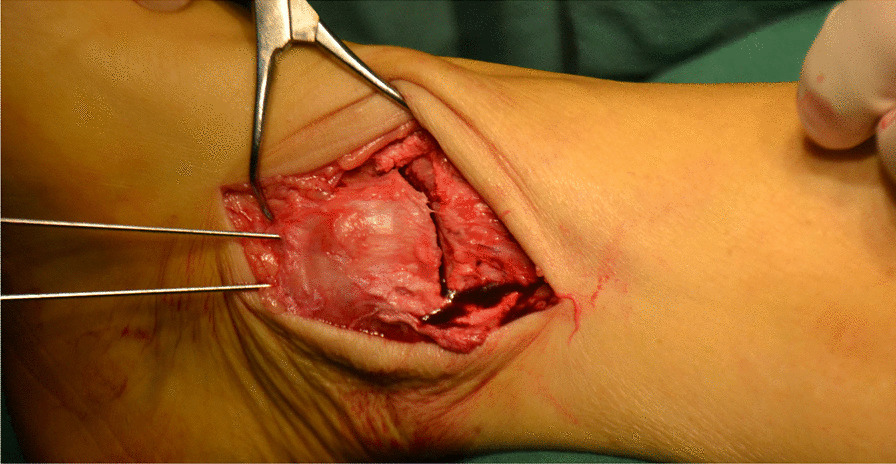
Reduction of medial malleolus osteotomy

**Fig. 13:**
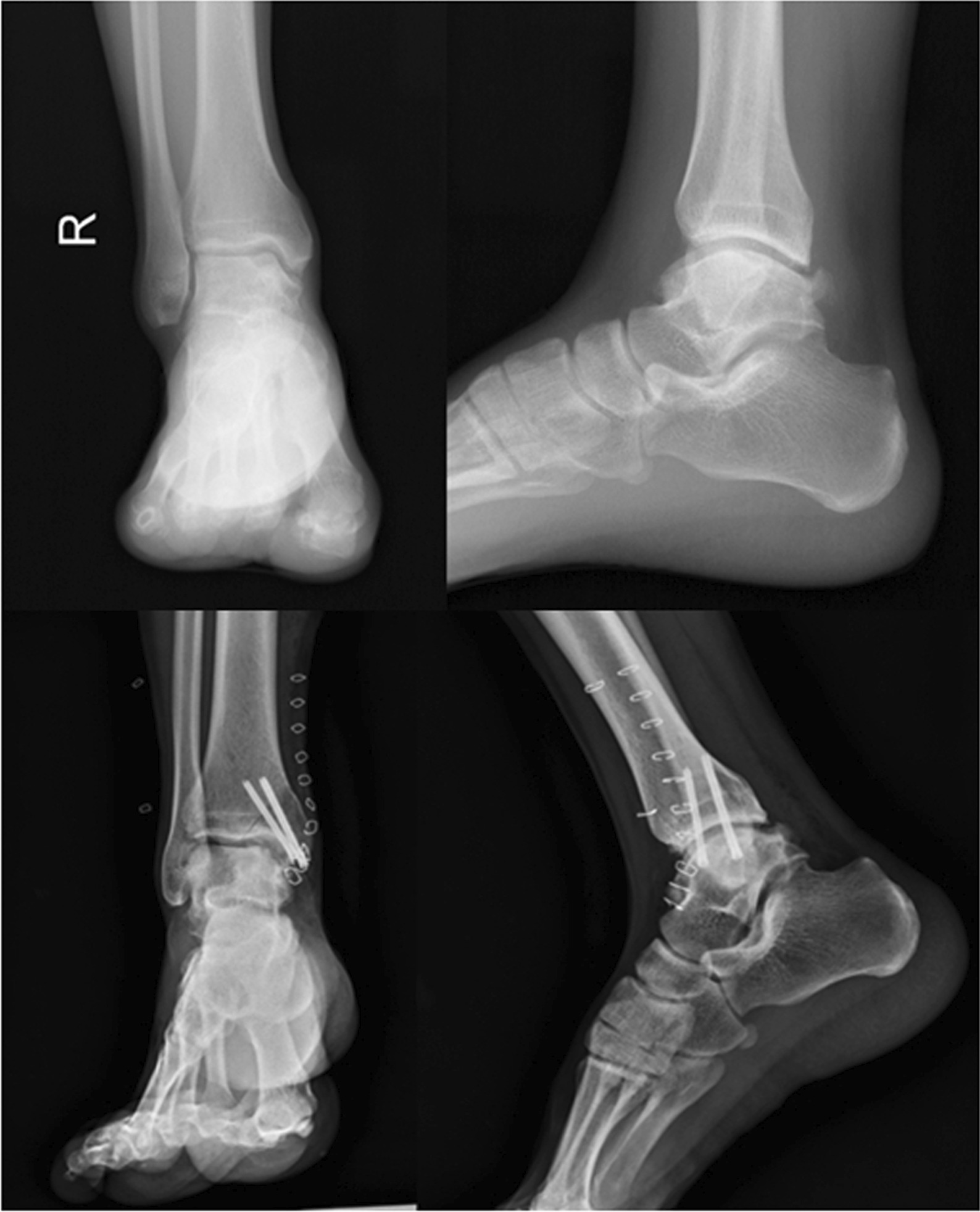
X-ray comparison before and after surgery

**Fig. 14:**
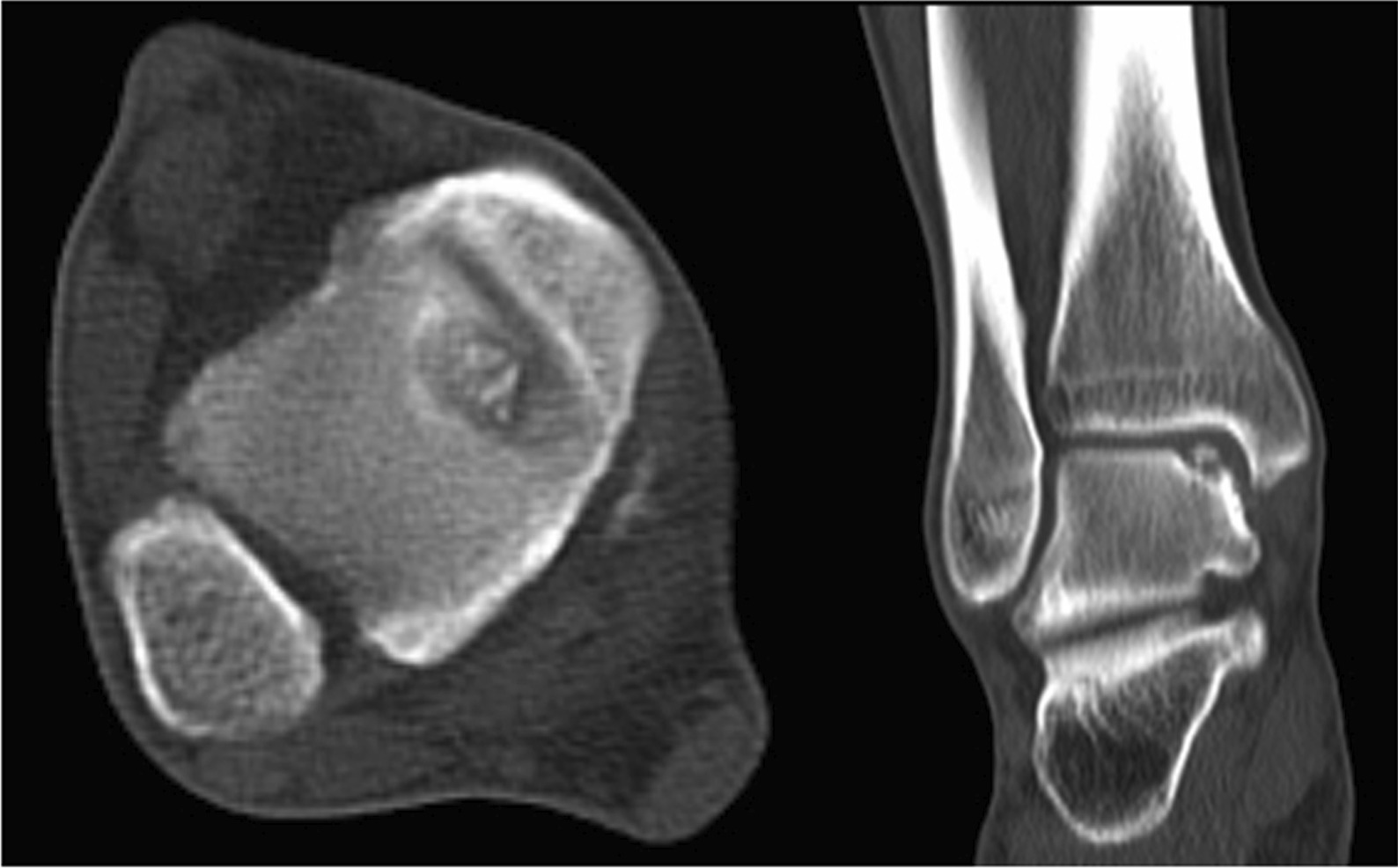
Preoperative CT shows medial talus necrosis

**Fig. 15:**
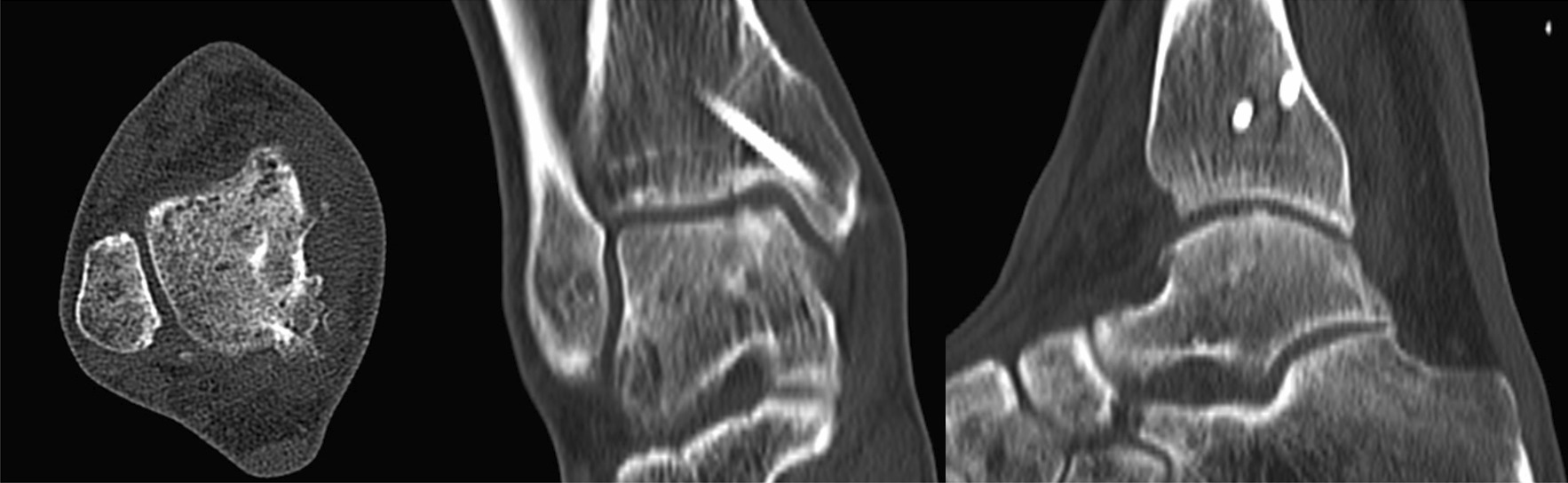
Postoperative CT shows medial malleolus osteotomy and talus bone graft have healed

All surgeries were performed under lumbar anesthesia with the patient in supine position and a sterile pneumatic tourniquet applied on the proximal thigh of the affected limb. The skin was routinely disinfected with iodine and alcohol and draped with a sterile surgical sheet before a 5-cm-long curved incision was made on the anteromedial side of the affected ankle. Two 3-mm-diameter Kirschner wires were used to enter the tibia at a 45° angle, and about 2 cm above the tip of the medial malleolusone, one 2.5 mm Kirschner wire was used to enter the tibia and remained vertical to the previous Kirschner wire. X-ray fluoroscopy showed that the position was satisfactory. The swing saw was used to cut off the medial tibia along the direction of 2.5 mm Kirschner wire, the valgus ankle joint fully exposed the cartilage damage area on the medial side of talus fornix, and the cartilage scraper was used to remove the degraded cartilage. After assessing the size of the lesion, a bone extractor with a suitable diameter was selected to punch a vertical hole in the joint surface and remove the lesion and cyst. Kirschner wire was used to make microfractures in the sclerotic bone around the cystic cavity, and take uniform blood infiltration as the standard. The incision of the anterior superior iliac spine on the same side is about 2.5 cm long. The bone extractor is used to remove the iliac composite bone column. When taking out the bone, pay attention to avoid damaging the inner and outer walls of the iliac bone. After trimming the damaged area to an appropriate size, plant it on the damaged part of the talus cartilage, and trim the filling area to ensure that the graft is flush with the surrounding joints. During the reduction of the medial malleolus osteotomy block, follow the bone guide tunnel reserved before the 3.0 mm Kirschner wire, use the 4.0 mm hollow drill to expand the bone tunnel, use the half thread hollow lag screw to pass through the Kirschner wire to fix the osteotomy site, remove the Kirschner wire, and conduct X-ray fluoroscopy to confirm that the reduction of the osteotomy site is satisfactory, the bone cartilage graft in the graft area is fully filled, the joint surface is restored to be flat, and the incision is closed layer by layer (Figs. 1, 2, 3, 4, 5, 6, 7, 8, 9, 10, 11, 12, 13, 14, 15).

### Postoperative rehabilitation

Postoperative, all patients used the same rehabilitation training method and was given plaster external fixation of lower limbs for 45 days; on postoperative day 2, patients began isometric muscle contraction and interphalangeal joint flexion and extension activities on their own. After 2 weeks, after suture removal, patients began plantar flexion, dorsiflexion, inversion, and valgus activities of the ankle. After one month, patients began partially weight-bearing with crutches. After 6 weeks, remove the plaster and gradually increase the load to full load, but prohibit vigorous activities. After three months, patients were permitted to walk with full weight-bearing and gradually return to normal life.

### Outcome measures

All patients were assessed by independent investigator preoperatively and at 3 and 24 months postoperatively. Routine radiologic examination comprised anteroposterior and lateral radiographs or CT of the ankle. To observe the bone healing at the osteotomy of medial malleolus and the bone healing of grafts in the injured area of talus cartilage. Due to the existence of metal internal fixation in the patient's ankle, the patient did not undergo MRI after operation. At the last follow-up, the AOFAS ankle hindfoot score and the VAS score were used to evaluate the patient’s ankle joint function and pain. At the same time, the patient’s iliac bone extraction site was monitored for surgical incision healing, whether there was pain, and whether there were other possible complications. To reduce errors and ensure the accuracy of data, all measurements were independently performed by three investigators, and the average of the three results for any measurement was used in the analysis.

### Statistical analysis

The statistical analysis was performed using the data analysis program SPSS Statistics version 24.0 software (version 24.0 for Windows; SPSS, Inc., Chicago, IL, USA). The paired t test and Wilcoxon’s signed-rank test were used to compare pre- and postoperative values (AOFAS ankle hindfoot scores, VAS scores, complications). Statistical significance was accepted for *P* values < 0.05.

## Results

Postoperatively, all patients were followed up for 28 (range 24–36) months. In 19 patients, the symptoms of pain around the ankle joint and functional limitation were significantly improved. In 11 patients, the pain symptoms basically disappeared, and the ankle joint activities basically returned to normal. Only 2 patients had poor postoperative results, which will be described in detail later. All patients underwent ankle X-rays and CT results after surgery. The results showed that the osteotomy area was reduced satisfactorily, the fixation was firm, and the bone graft was well filled. The imaging examination 3 months after operation showed that all patients achieved bone healing in osteotomy area and bone graft area.

### Ankle Function Score

The AOFAS scores of patients were (49.7 ± 8.1), (80.4 ± 3.6) and (89.2 ± 6.4), respectively, preoperative, 3 months after operation and at the last follow-up. There was significant difference between preoperative AOFAS score and 3 months after operation (*P* < 0.05), and there was also significant difference between 3 months after operation and the last follow-up (*P* < 0.05). The AOFAS score showed that the patient's daily activity function of the ankle joint was significantly improved at 3 months after surgery, and at the last follow-up, the ankle joint function was better than 3 months after surgery.

### Ankle Pain Score

The VAS scores of patients were (6.2 ± 1.7), (2.1 ± 0.9) and (1.5 ± 0.8), respectively, preoperative, 3 months after operation and at the last follow-up. There was a statistically significant difference between 3 months after operation and before operation (*P* < 0.05). However, there was no statistically significant difference between 3 months after operation and the last follow-up (*P* > 0.05). This result shows that the patient's ankle pain symptoms have been significantly reduced at 3 months after surgery, and the surgical effect is satisfactory.

### Complication

In the 3 months postoperative and the last follow-up, the surgical incision in the iliac donor area healed well, and no patient had pain or swelling in the ipsilateral iliac donor area. The tibial osteotomy area and the talar bone graft area did not have any complications such as bone nonunion, incision infection or poor healing, ankle instability, and there was no loosening or falling off of the internal fixation.

Two patients were not satisfied with the postoperative effect. One patient was dissatisfied due to severe cicatricial diathesis; after the surgical incision healed, the scar gradually increased, resulting in medial soft tissue contracture and limited foot valgus movement. The patient was treated with extracorporeal shock wave therapy (Switzerland, ENS), with each impact administered 2000 times (8 Hz, 2.0–3.0 bar), 7 days comprising one cycle, and four to eight cycles comprising one treatment course. After six treatment courses, the ankle mobility was restored but still mildly limited, with a mobility of 28.1°. The other patient who was dissatisfied with the result had undergone a rapid increase in bodyweight from 71 kg (BMI 23.7 kg/m^2^) preoperatively to 98 kg (BMI 32.7 kg/m^2^) at final follow-up. The patient did not complete the rehabilitation training, which led to ankle stiffness that prevented them from returning to their previous level of sports activity. However, the preoperative ankle function was achieved. After 2 months of correct guidance and physiotherapy, the ankle stiffness had markedly improved.

## Discussion

OLT is very common in clinical practice and is increasingly affecting patients. Various surgical treatment regimens have been used in clinical practice, but the optimal option remains unclarified [1]. Especially, the talar cartilage injury of Hepple V is a great challenge for many doctors. The present study retrospectively analyzed 32 patients with Hepple V osteochondral lesions of the talus with subchondral cysts treated with iliac bone autografting. The results suggest that this surgical approach for OLT achieves significant improvements without serious complications. and they are very willing to undergo the same procedure if a similar disease occurred on the other side.

There are multiple treatment approaches for Hepple stage V OLT with subchondral cysts, and the optimal surgical procedure remains controversial; however, the clear goals of treatment are to repair the OLT, remove the subchondral cyst, and restore the articular cartilage surface [19]. Autologous and allogeneic osteochondral grafting are reasonable options for repairing OLT with cysts. When the area of osteochondral injury is large and many autologous bone fragments are required to fill the osteochondral lesion, allogeneic bone grafting is preferred; however, allogeneic bone grafting has many limitations, including the risks of rejection and disease transmission, and the high cost due to the limited number of donors [8, 9]. And Migliorini found talar osteochondral transplant using allografts was associated with higher rates of failure and revision compared with autografts at midterm follow-up by 40 studies (1174 procedures) with a mean follow-up of 46.5 ± 25 months were retrieved [20]. However, there is no immune rejection in autologous bone cartilage transplantation, and fresh autologous grafts can improve the bone healing rate, Valderrabano et al. [21] reported that autologous osteochondral transplantation of knee cartilage to treat talar lesions resulted in considerable complications, swelling, and strangulation in the operated area of the knee in the 12 of 21 patients who completed 72 months of follow-up postoperatively. Thus, the indications for mosaicplasty with a plug transfer from the knee to the talus must be considered carefully. In addition, there are significant anatomical and biomechanical differences between the knee and ankle, and the healing rate after grafting knee cartilage to talar cartilage is unclear [8, 9]. Moreover, there may be differences in the composition of cartilage matrix in the non-weight-bearing area of knee joint and talus cartilage, which may also lead to the recurrence of ankle pain and ankle dysfunction.

Iliac bone autografting for medial OLT with subchondral cysts does not damage other joints, repairs the degenerative talar tissue, and fills the bone cyst. The iliac bone contains a large amount of cancellous bone, which is rich in blood supply. It achieves good postoperative bone healing. At the same time, the internal and external walls of the iliac bone will be preserved during bone removal to ensure the integrity of the iliac bone as much as possible. Furthermore, the periosteum of the iliac bone contains stem cells that differentiate into chondrocytes and fibrocartilages, and the iliac bone and periosteum are tightly integrated at the cellular and extracellular levels, leading to the formation of a whole graft with intrinsic mechanical stability and biological interaction [22]. The periosteum–iliac bone autografting procedure is technically mature, with few complications, and sufficient graft bone can be obtained [23].

In this study, when the surgical area was exposed, the medial malleolus was osteotomized, the ankle valgus, the cartilage injury area could be fully exposed, and the subchondral cyst could be completely removed. The preoperative assessment of the size of the subchondral cyst and whether it is completely removed during the operation is closely related to the success rate of the operation. Therefore, the cyst must be completely removed before the cartilage transplantation to reduce the probability of nonunion of the postoperative bone graft. In this study, a bone extractor was used to completely remove the talus necrosis bone, and Kirschner wires were used to create microfractures in the sclerotic bone around the cyst cavity. Taking uniform blood infiltration as the standard, it was ensured that the transplanted bone had sufficient blood supply to promote the healing process. Using the bone extractor to remove the iliac bone composite column can also avoid excessive damage to the iliac bone. After removing the iliac bone graft, compare it with the talus necrosis bone, trim the graft bone, and ensure the sufficient osteochondral graft and the flatness of the articular surface. Kirschner wire was used as the guide of the fixation screw to achieve the anatomical reduction of the medial ankle after operation. At the last follow-up, 32 patients did not find nonunion at the osteotomy, and the bone graft and osteotomy healed well.

This study had some limitations. Postoperative MRI can monitor the repair of the talar cartilage, but due to the presence of metal internal fixation, the patient’s MRI was not examined. The study design was retrospective, there was no control group for comparison, and the sample size was small. Further research is warranted to confirm the present findings.

## Conclusions

Iliac bone autografting for medial OLT with subchondral cysts restored the talar cartilage defect, effectively relieved pain, and significantly improved the ankle function and quality of life.

## Data Availability

The patients’ data were collected in the Third Hospital of Hebei Medical University. The datasets used and/or analyzed during the current study are available from the corresponding author on reasonable request.

## Abbreviations

OLT: Osteochondral lesion of the talus
VAS: Visual Analogue Scale
AOFAS: American Orthopaedic Foot & Ankle Society
